# Treatment Seeking Behaviors and Associated Factors among Patients Experiencing Acute Coronary Syndrome Using Health Belief Model in Addis Ababa, Ethiopia

**DOI:** 10.4314/ejhs.v32i4.15

**Published:** 2022-07

**Authors:** Lemlem Beza, Bekele Alemayehu, Adamu Addissie, Aklilu Azazh, Rebecca Gary

**Affiliations:** 1 Department of Emergency Medicine, College of Health Sciences, Addis Ababa University, Addis Ababa, Ethiopia; 2 Department of Internal Medicine, College of Health Sciences, Addis Ababa University, Addis Ababa, Ethiopia; 3 Addis Ababa University, College of Health Sciences, Addis Ababa, Ethiopia; 4 Nell Hodgson Woodruff School of Nursing, Emory University, Atlanta, Georgia

**Keywords:** Treatment seeking behavior, acute coronary syndrome, Ethiopia

## Abstract

**Background:**

Acute coronary syndrome (ACS) is a life-threatening condition. The mortality rate will be reduced if immediate treatment is provided. Patients' awareness of ACS is limited, so they do not seek help as quite often as they should. The level of treatment seeking behavior and associated factors among ACS patients admitted to three hospitals in Addis Ababa, Ethiopia, were assessed using a health belief model.

**Methods:**

A cross-sectional study was conducted among 330 ACS patients from November 2019 to December 2020. Sociodemographic and clinical variables data were extracted using pre-tested checklist. The outcome and other variables data were collected using the checklist and structured questionnaire. The data were entered into Epi-data 3.1 and exported to STATA 17.1 for analysis. Descriptive statistics relevant to the variable was performed. A multivariable logistic regression was used to identify factors associated with treatment seeking behavior.

**Results:**

This study revealed that the mean time from symptom onset to arrival at the emergency unit (EU) was 24 ± 19.5 hours, slightly < half of the participants (n=149, 45.1 %) had adequate treatment seeking behavior. Perceived threat (AOR=1.03,95% CI:1.01–1.06, p=0.002), perceived benefits (AOR=1.09, 95%CI: 1.02–1.0, p≤0.001), self-efficacy (AOR=1.16, 95% CI :1.01- 1.22, p≤0.001), education (AOR=2.2,95%CI:1.31–3.9, p≤0.01) self-autonomy (AOR=3.1,95%CI:1.82–5.4, p<.001) and no depression (AOR=1.9,95%CI:1.1–3.3, p≤0.05) were found to have significantly association with adequate treatment seeking behavior.

**Conclusion:**

This study indicates, less than half of ACS patients had adequate treatment seeking behavior. Thus, context-specific behavioral interventions, along with public awareness campaigns about ACS, should be implemented.

## Introduction

Cardiovascular disease (CVD) is the leading cause of morbidity and mortality worldwide with acute coronary syndrome (ACS) contributing significantly to the rising numbers in low-and-middle income countries (LMICs)(1). Studies in LMICs including Ethiopia indicate that up to 42% of premature deaths are related with CVD (2). The finding of a recent study shows the rising and emerging burden of CVD with ACS as one of the most prevalent non-communicable diseases in Ethiopia (3). Acute coronary syndrome is a range of conditions resulting in sudden interruption or reduction of coronary blood flow resulting in myocardial ischemia and infarction (4).

Optimal patient outcomes are associated with early initiation of treatment within 90 minutes of symptom onset (5). It is estimated that for every minute of delayed treatment beyond 90 minutes there is a proportional increase of 7.5% in one-year mortality (6). Delayed time to treatment also diminishes the benefits of recommended interventions such as thrombolytic therapy and percutaneous intervention (PCI) (7). Extended delay in treatment is linked to higher adverse cardiac events including cardiac arrest and sudden death (8). The reasons for delayed treatment seeking behavior for ACS symptoms are multifaceted and complex (9,10). Treatment seeking behavior (TSB) is defined as a decision for action or inaction by the individual who perceives themselves as having a health problem (11). Broadly, TSB describes health behavior reflected by activates undertaken to maintain good health or prevent the occurrence of ill-health (12). Treatment seeking behavior that begins as soon as symptoms appear is crucial for better ACS outcomes. Studies suggest that the time from ACS symptom onset to treatment is often delayed due to a lack of awareness or incorrect interpretation of symptoms (13). In addition, resources to provide early treatment are often lacking in LMICs including emergency medical response systems and cardiovascular centers with intervention capabilities.

Therefore, the purpose of this study was to examine the treatment seeking behaviors and associated factors in patients with a confirmed diagnosis of ACS admitted to emergency units (EU) at three hospitals in a large urban setting in Ethiopia using HBM.

## Methods and Materials

**Study design, and setting**: An institutional based cross-sectional study was conducted in Addis Ababa, Ethiopia, among confirmed ACS patients admitted to the EUs of two public tertiary hospitals and a private cardiac center. These three hospitals were chosen because they see a large number of cardiac patients seeking CVD services, and also provide advanced cardiac care services such as angiography, reperfusion therapy, and percutaneous coronary intervention.(PCI)(14). Furthermore, the chosen facilities had an established coronary care unit, emergency admission unit, emergency and critical care physicians, and cardiologists for managing acute cardiac emergencies such as ACS. Data were collected from November 2019 to December 2020.

**Eligibility criteria**: Patients with a confirmed diagnosis of ACS who were 18 years old or older were recruited to take part in the study. Abnormal cardiac bio-markers such as elevated cardiac enzymes (Troponin -I Creatine Kinase -MB (CK - MB), electrocardiogram (EKG), or symptoms consistent with ACS (chest pain, shortness of breath, dizziness, or light-headedness) if there is >1–2mm of ST elevation in two contiguous leads on the ECG or new LBBB and Cardiac Troponin I elevated for patients with STEMI, ST-segment depression or T-wave inversion combined with elevated troponin I for NSTEMI, chest pain without ECG changes and no troponin I elevation for unstable angina

In addition, the participant's recalling the onset of symptoms and events prior to admission to the hospital. Patients were eligible to participate in the study if they were hemodynamically stable and had waited for 48–72 hours until being free of chest pain or discomfort at the time of the interview.

**Exclusion criteria**: Those with cognitive impairment or unable to understand or communicate clearly or appropriately, psychiatric disorder, critically ill, mechanically ventilated, participants diagnosed AMI after admitted in the EU, terminal illness, multiple diagnoses that complicated symptom recognition and refusal to participate in the study were excluded.

**Sample size and sampling**: All the consecutive ACS patients included the required sample size was estimated based on two population proportion formulas using StatCalc of Epi-info version 7.2.2.6 statistical software. The proportion of acute coronary syndrome patient with pain reaction (28.6%) and Odds ratio of 1.91 were used to calculated the required sample(15). In addition, we used 5% of level of significance and power of 0.8(16). Accordingly, a total of the 375 sample of ACS patients was estimated.

**Data collection instruments**: Data were collected using a psychometrically sound instrument adapted from a previous study. In multiple studies, the majority of the instruments were translated into Amharic and validated in an Ethiopian population includes ; Heath Belief Survey (HBM-Survey) (17), Patient Health Questionarree-2 (PHQ2)(18) and the ENRICHD social support instrument (ESSI) (19). However, the Acute Coronary Syndrome Response Index (ACSRI) questionnaire had never been tested in an Ethiopian population. An expert translated this instrument, which was originally written in English, into Amharic (Ethiopian local language).

In addition, the ACSRI was pilot-tested in 10 non-study participants. It was slightly modified to improve readability and simplicity. The ACSRI was used to measure knowledge, attitudes, beliefs and treatment seeking behaviors for ACS(20), and was used widely in ACS patients. Treatment seeking behavior scale has two items, which measure the level of treatment seeking behavior during the ACS episode. Participants who scored greater than or equal to the mean score were considered as having adequate treatment-seeking behavior. The HBM questionnaire has five subscales and, all the items of the subscale have the following five -point scale response choice; strongly disagree (1 point); disagree (2-point); neutral (3-points); agree(4-ponts); and strongly agree (5-points).

The score for each sub-scale was assessed separately. The higher the subscale score indicated a higher probability of individual beliefs. Higher perceive barriers to TSB however, indicated the opposite with higher scores indicating inability to overcome TSB. Cronbach's alpha coefficient for the original HBM was 0.73 to 0.79 (Champion, 1984). The reliability coefficient for the constructs of health belief model for the current study were 0.65, 0.79, 0.89, 0.93, 0.95 for barrier, self-efficacy, benefit, seriousness and susceptibility for acute coronary syndrome, respectively. Patient Health Questionnaire (PHQ-2) it is 2-item questionaries were used to measure frequency symptoms of depression. Participants are asked to rate their symptoms of depression over the past two weeks concerning “little interest or pleasure doing things and feelings of being depressed or hopeless”.

The responses are rated in a Likert scale format with 0 reflecting not at all to 3 representing nearly every day. The scoring for the PHQ2 ranges from 0–6 with higher scores representing increased depressive symptoms(21). ENRICH Social support instrument (ESSI), used to measure the participants range of social support across the life time. The ESSI was “originally developed to assess social support among post-myocardial infarction (22). The Cronbach alpha for the present study was 0.98 among ACS patients. It is a five item self-report questionnaire which rates on a 5-point Likert scale 0 meaning none of the time, 5 means all of the time.

**Data collection procedure**: We recruited a team of three registered nurses from each study site who work at the respective hospitals' EU as data collectors. Prior to data collection, they received three days of training on the objective, methods, conducting an interview, and abstracting data from patient charts. Furthermore, in order to facilitate ACS patient access, EU coordinators, senior emergency physicians, and residents communicated and were briefed on the study. Primary and secondary data were collected from ACS patients and their charts using a structured questionnaire and data extraction checklist.

**Data entry and analysis**: To reduce data entry error, data were coded and entered into Epi-data 3.1 before being exported to the STATA 17.1 statistical package for analysis. Prior to data analysis, data from the patient chart and interviews were checked for accuracy and completeness. Box plots were used to assess the distribution of continuous data. For continuous variables, descriptive statistics were used, and for other variables, frequency (%) was used. Multivariable logistic regression was used to identify factors associated with treatment seeking behavior. The crude and adjusted odds ratios were calculated, along with the corresponding confidence interval (CI). The level of significance was set at 0.05.

**Ethics approval and consent to participate**: The Institutional Review Board (IRB) of Addis Ababa University's College of Health Sciences granted ethical approval with IRB number (No.078/19/Nursing). The study's purpose, general content was explained in the study participant's preferred language, and informed consent was obtained from all participants. The participants were informed that they had the freedom to participate in the study or not, and the right to withdraw at any time during the interview.

## Results

**Demographic and clinical characteristics**: Of the total 375 patients diagnosed with ACS, 330 agreed to participate in the study with overall response rate of 88%. Majority of the respondents (n=219, 66.3%) were males, and the mean age was 57.9 ± 14.1 years, (n= 301; 92.2%) were married and (n=228, 69%) resided in an urban area. Hundred thirty-eight (41.8%) of the participants were attended college diploma and above and 185 (56%) participants had diabetes, 72.9 % (n =198) sever pain (Table 1).

**Table 1:** Socio-demographic characteristic ACS patients admitted at public and private hospital of Addis Ababa, Ethiopia (2019–2020) (n=330)

Variable	n (%)	M(SD)
Gender		
Male	219 (66.3)	
Female	111(33.6)	
Age		57.9(14.1)
Marital status		
Never married	29(8.7)	
Ever Married	301(92.2)	
Place residence		
Urban	228 (69)	
Rural	102(30.9)	
Education		
No formal education	80(24.2)	
Primary/secondary	112(33.9)	
College diploma & above	138 (41.8)	
Occupation		
Employed	138(41.8)	
Unemployed	135(40.9)	
Retired	57 (17.2)	
History		
Diabetes	185(56)	
Hypertension	180(54.5)	
Smoking	71(21.8)	
Level of chest pain		
Mild pain	22(8)	
Moderate pain	52(19.1)	
Sever pain	198 (72.9)	

**Psychosocial response to ACS symptoms**: Two hundred eighty-one (85%) relied on non-ambulance transportation to reach the hospital. And also, 212 (64.24 %), were presented in EU 12 hrs or more after the onset of ACS symptoms (Table 2).

**Table 2:** Psychosocial and other factors related with treatment seeking behavior ACS patients admitted at public and private hospital of Addis Ababa, Ethiopia (2019–2020) (n=330)

Variables	Frequency	Percent
Hospital type		
Private	182	55.1
Public	148	44.8
Mode of transport		
Ambulance	49	14.8
Non-ambulance	281	85.1
Who decided to seek help		
Myself	175	53
aOther family members	155	47
Pre-hospital delay		
<12 hours	118	35.7
>=12 hours	212	64.2
Social support		
Inadequate	191	57.8
Adequate	139	42.1
Depression symptoms		
No depression symptoms	141	42.7
Depression symptoms	189	57.2

a Other family members; Husband; wife; Elder child; My mother; Neighbor

Type of diagnosis treatment or intervention provided for ACS patients: The majority of the participants were diagnosed as having ST-segment elevation myocardial infarction (STEMI) (n=161, 48%) followed by non-ST-segment elevation myocardial infraction (NSTEMI) (n=122,36.9). Angiography was performed for STEMI (n=93,58.1%) and NSTEMI (n=42, 34.4%) (Table 3).

**Table 3:** Type of ACS, intervention/treatment provided who were admitted in private and public hospitals of Addis Ababa, Ethiopia from 2019/20 year

Drugs	Type of ACS			
	Unstable angina frequency n=47, (14.2%)	NSTEMI frequency n=122 (36.9%)	STEMI frequency n=161 (48.7%)	Total frequency (%)
Angiography	14(29.79)	42(34.43)	93(58.13)	149(45.29)
PCI	5(10.64)	31(25.41)	83 (51.55)	119(36.06)
CABG	0(0.00)	2 (1.64)	7(4.35)	9(2.73)
Streptokinase	0(0.00)	0(0.00)	2(1.30)	2(0.63)
Aspirin	47 (100)	121(99.1)	161(100.0)	329(99.6)
Clopidogrel	42 (89.36)	111 (90.98)	151 (93.79)	304(92.12)
Beta blockers	14(30.43)	40(34.7)	54(35.53)	108(32.70)
ACE inhibitor	13(26.6)	37 (30.33)	53(32.9)	103(31.21)
Statin	43 (91.49)	113 (92.62)	158 (98.14)	313(95.15)
Proton-pump-inhibitor (PPI)	31(65.96)	78(63.93	132(81.99)	241(73.03)
Anticoagulants	18 (38.30)	41 (33.61)	68 (42.24)	127 (38.48)
Antipain	20 (55.56)	49 (50.00)	39 (32.77)	108 (42.69)

STEMI; ST-elevation myocardial infarction, NSTEMI; Non-ST elevation myocardial infarction, UA: unstable angina, PCI; percutaneous intervention, CABG; Coronary artery bypass grafting, ACEI; Angiotensin-converting enzyme inhibitors

Treatment Seeking Behaviors of ACS patient: The mean score of treatment seeking behavior ACS patients was 4.4 and those who scored greater than or equal to the mean score were considered as having adequate treatment-seeking behavior. The current study indicated that more than half of the participants (n=181, 54%) had inadequate treatment seeking behavior for ACS symptoms while one hundred forty-nine (45%) of the participants had adequate treatment seeking behavior.

**Factors associated with level of treatment seeking behavior**: Candidates for multivariable logistic regression were selected in bivariable analysis with p-values <=0.25. First, we examined the predictive values of the pre-existing variables (Model 1) which revealed that some of the pre-existing variables contributed for 30% of the variance in adequate treatment seeking behavior. (Model 2) Upon adding all of the health belief model -variables in the model, the results showed that model 2 explained 40% of the variance in adequate treatment seeking behavior. (Table 4). In **Model 1** Those respondents who attended college and above education were 5.7 times more likely to have adequate treatment seeking behavior as compare with those illitrate (AOR = 5.7, CI : 2.7–11.6, p=<0.001). ACS patients who made self-decision were 3.0 times more likely to have adequate treatment seeking behavior as compare with those whose decision made by other family member (AOR=3.0, CI:1.7–5.1, p=<0.001). ACS patients who had exposure to media were 1.7 times more likely to have adequate treatment seeking behavior as compare with those who had not(AOR=1.7, CI:1.01–2.8, p=0.044). ACS patients who get adequate social support were almost 2 times more likely to have adequate treatment seeking behavior as compare with those did not get adequate social support (AOR=1.96, CI:1.14 - 3.3, p=0.014). **In model 2**, ACS patient who perceived benefit were 1.1 times more likely to have adequate treatment seeking behavior as compare with those who did not (AOR=1.09, CI:1.02–1.0), ACS patient who had self-efficacy were 1.1 times more likely to have adequate treatment seeking behavior as compare with those who had not (AOR=1.1, CI: 1.01 – 1.22). ACS patients who perceived threat (AOR=1.02, CI: 1.01 – 1.05) were 1.02 times more likely to have adequate treatment seeking behavior as compare with those who did not. Conversely, higer perceived barrier (AOR= 0 .91, CI: 0.86 –.0.97) were associated with inadequte treatment seeking behaviour (Table 4).

**Table 4:** Factors associated with treatment seeking behavior in ACS patients admitted at public and private hospital, Addis Ababa, Ethiopia (2019–2020) years

Variable	Model 1^a^, AOR with (95% CI)	Model 2^b^, AOR with [95%CI]
Preexisting variables		
Level of education	Base	Base
Illiterate		
primary/secondary education	2.08[1.01–4.27]*	-----
College and above	5.7 [2.7–11.6]***	2.2 [1.31–3.9] **
Decision by		
myself	3.0 [1.7–5.1]***	3.1 [1.82–5.4] ***
Other family	Base	Base
Exposure to mass-media		-----
Radio	1.7 (1.01–2.8)*	
Depression		
No	1.6 [.98–2.8]	1.9 [1.1–3.35] *
Yes	Base	Base
Social support		-------
Adequate	1.96 [1.17–3.2]*	
Inadequate	Base	
Knowledge		----
Adequate	1.6 [0.96–2.7]	-----
Inadequate	Base	
Model variable		
Perceived threat		1.02[1.01–1.05]*
Self-efficacy		1.1 [1.01–1.22]*
Barriers		.91 [0.86–.0.97]**
Benefit		1.09[1.02–1.0]**

* p<.05

** p<.01

*** p<.001

Model 1: ^a^ multiple regression of preexisting variables with outcome

Model 2: ^b^ Multiple regressions of preexisting and model variables with outcome

AOR; adjusted odds ratio is reported and CI, confidence interval in the parentheses

## Discussion

The health belief model was used to assess the level of treatment seeking behavior of patients admitted to the emergency departments of three hospitals with a confirmed diagnosis of ACS. The health belief model used as the study's guiding framework. The framework's explanation for use is that it explores the effects of pre-existing conditions on individual's beliefs that lead to action or inaction. The model's five health belief constructs, such as perceived threat (susceptibility and seriousness), greater perceived benefit, and self-efficacy of help seeking, were all found to be significantly associated to adequate treatment seeking behavior.

Accordingly, we found majority of participants had inadequate seeking behavior for ACS symptoms. Individual beliefs such as greater perceived threat, perceived benefit, and high self-efficacy were statistically significantly associated to adequate treatment seeking behavior in response to ACS symptoms. Nevertheless, Participants who were unable to overcome perceived barriers to treatment seeking behavior, had a lower educational status, were less autonomous, had insufficient knowledge about ACS, and had depressive symptoms were associated with inadequate treatment seeking behavior. The present study suggested that increasing awareness of ACS symptoms, as well as context-appropriate behavioral interventions focusing on those at risk, are required for appropriate and timely treatment seeking behavior aimed at reducing adverse outcome.

Many of our findings regarding sociodemographic and clinical characteristics of the ACS participants are similar to other studies conducted in Ethiopia and other LMICs. The majority of ACS patients were younger in age compared to high income countries where the average age of ACS is 72 years(23). Most participants were diagnosed with ST segment elevation myocardial infarction (STEMI) and similar to previous reports in both high and LMICs (24, 25).

In the present study, the time from ACS symptom onset to treatment was 12 hours or longer and is supported by Bogale and colleagues(24), from Ethiopia and in other LMICs(26, 27). The association between delayed treatment in ACS in LMICs is well established (28–31). Lack of awareness of symptoms as cardiac in origin particularly among those with lower sociodemographic, educational status and female gender were the factors that contribute to increase in delay from symptom onset to treatment in LMICs(32, 33). Poorly developed emergency medical services and communication between community hospitals and urban cardiac centers have also been implicated as major reasons for treatment delays (34, 35).

The strong association between lower educational level and inadequate treatment seeking bahviour is well-established both in high and LMICs (36, 37). These findings are also consistent with a community study conducted in southern Ethiopia (38). The reasons for inadequate treatment seeking behaviur among those with lower educational level may be a lack of awareness about ACS symptoms or the required knowledge and skills to make effective and timely decisions.

Studies reported association between older age, female gender and marital status with inadequate treatment decisions (36, 37, 39). In the present study however, there were no significant associations between these sociodemogrhic variables such as gender, age, place of resdinence and treatment seeking behaviour and is also supported in previous work (40). The reasons for these differences are unclear but may be due to varying geographic locations, settings, sociocultural beliefs and measurment diffrence. The ability to correctly identify a cardiac symptom and self-decision to seek treatment was also associated with early descition to seek help (41).

This is underscored by the health belief model that predicts that individuals will take action to avoid or alleviate symptoms or illness when they perceive that they are susceptable to it and precived that there will be negative consequence from failing to take action i.e, the consequence may be worse (42). This finding is consistent with the result of other similar studies(43, 44). Perceived threat was significantly associated with adequate treatment seeking behavior, in which people who have an insight about the potential consequences of a disease will naturally seek treatment early.

Self-efficacy of help seeking behavior in response to ACS symptoms is also highly linked with taking early treatment action (45). The findings were similar with the finding of study by Brick E et al in which ACS patients with higher self-efficacy had adequate treatment seeking behavior and improved health-related quality of life after experiencing an AMI (46). High perceived self-efficacy is considered a strong influential factor in treatment seeking behavior since individuals must have confidence to organize themselves to seek certain goals. Studies show that individuals with a high level of perceived self-efficacy have a greater commitment to engage in activities at a time of challenges and difficulties, and spent more time and effort on activities such as seeking help during an AMI (38, 45, 47).

The other health belief model construct which is inversely associated with help seeking behavior in response to ACS symptoms is perceived barrier. In our study, participants who had difficulty to overcoming barriers of help seeking decisions such as (cost, transporation, time and lack of trust on the treamnet ) had inadequate treamnet seeking behaviour in response to ACS symptoms.. Other findings also support that higher perceived barriers are predictor of delayed treatment seeking behaviour (28, 29). In addition, participants who had depressive symptoms were also more likely to have inadequate treatment seeking for an AMI(48, 49). Depressive symptoms are well established to have negative cardiovascular effects, lower ability to participate in effective self- care and slow decision making that result in delayed response to symptoms. Screening for depressive symptoms using a simple tool such as the PHQ-9 may enable clinicans to more quickly identify those at higher risk for poor decision- making regarding symptom onset, severity and appropriate responses for early seeking for help.

The study indicates less than half of the participants had adequate treatment seeking behavior for ACS. Higher educational level, independently decided, high perceived threat of ACS, benefit and self-efficacy were found to have statistically significantly associated with adequate treatment seeking behavior. However, depressive symptoms and perceived barriers were associated with inadequate treatment behavior. The finding warrants an urgent need to design and implement culture and context specific behavioral intervention with an emphasis on the benefit of appropriate and early response during ACS episode. Moreover, public awareness campaigns about ACS, focusing those at risk along with improving nationwide accessibility of ambulance services.
